# Tuberculosis in found dead badgers at the edge of the expanding bovine tuberculosis epidemic

**DOI:** 10.1038/s41598-025-86930-y

**Published:** 2025-03-27

**Authors:** Siân M. Powell, Nicola Dessi, Malcolm Bennett, Belinda Wang, Andrew Robertson, Elisabeth Waller, Graham C. Smith, Richard J. Delahay

**Affiliations:** 1 National Wildlife Management Centre, Animal and Plant Health Agency, Stonehouse, GL10 3UJ UK; 2 School of Veterinary Medicine and Science, University of Nottingham, Loughborough, LE12 5RD UK; 3 Department for Environment, Food and Rural Affairs, 1a Page Street, London, SW1P 4PQ UK; 4 Department of Epidemiological Sciences, Animal and Plant Health Agency, Weybridge, Addlestone, KT15 3NB UK

**Keywords:** Microbiology, Ecological epidemiology

## Abstract

Bovine tuberculosis (bTB) is a major disease of cattle in the UK, placing a significant economic burden on the taxpayer. The causative agent, *Mycobacterium bovis*, has a wide host range, including the European badger (*Meles meles)*. While badgers have been implicated in the transmission and maintenance of infection in cattle in areas of endemic disease, their role at the edge of the endemic area is poorly understood. Here we present data on the prevalence of infection in badgers collected along the southern edge of England’s bTB epidemic. Stakeholders across five counties (Oxfordshire, Berkshire, Buckinghamshire, Hampshire, and East Sussex) submitted found-dead badgers for post-mortem examination and testing by bacterial culture. The overall prevalence, as confirmed by whole genome sequencing, was 6.5% (28/428), ranging between 1.1% (1/88) in Hampshire and 13.0% (14/108) in Oxfordshire. The commonest *M. bovis* clade in badgers was B6-62, which was predominant in 4/5 counties. B6-62 was also the commonest clade found in cattle and was detected in all counties except East Sussex where, although absent from the cattle population, it was detected in local badgers. This study highlights the co-incidence of infection in badgers and cattle in parts of the southern edge area consistent with localised clustering of infection in both species.

## Introduction

*Mycobacterium bovis* (*M. bovis*) is a member of the *M. tuberculosis* complex (MTC), a group of host-associated, tuberculous disease-causing bacteria of high genetic similarity^1,2^. Despite this high level of clonality, the group varies significantly in host tropism with *M. bovis* having the widest known host range^3^. Indeed, in the United Kingdom (UK) alone, *M. bovis* has been detected in nine species of farmed or companion mammals, and at least 20 species of wild mammals^4–9^. In cattle, *M. bovis* causes bovine tuberculosis (bTB), one of the most significant challenges affecting cattle health in England, which is estimated to cost the UK taxpayer over £150 million per annum, with further costs to the cattle industry^10^. In addition to the financial burden is the cost of livestock life, with over 31,000 cattle slaughtered in 2023, of which 20,200 were from England^11^.

**Fig. 1:**
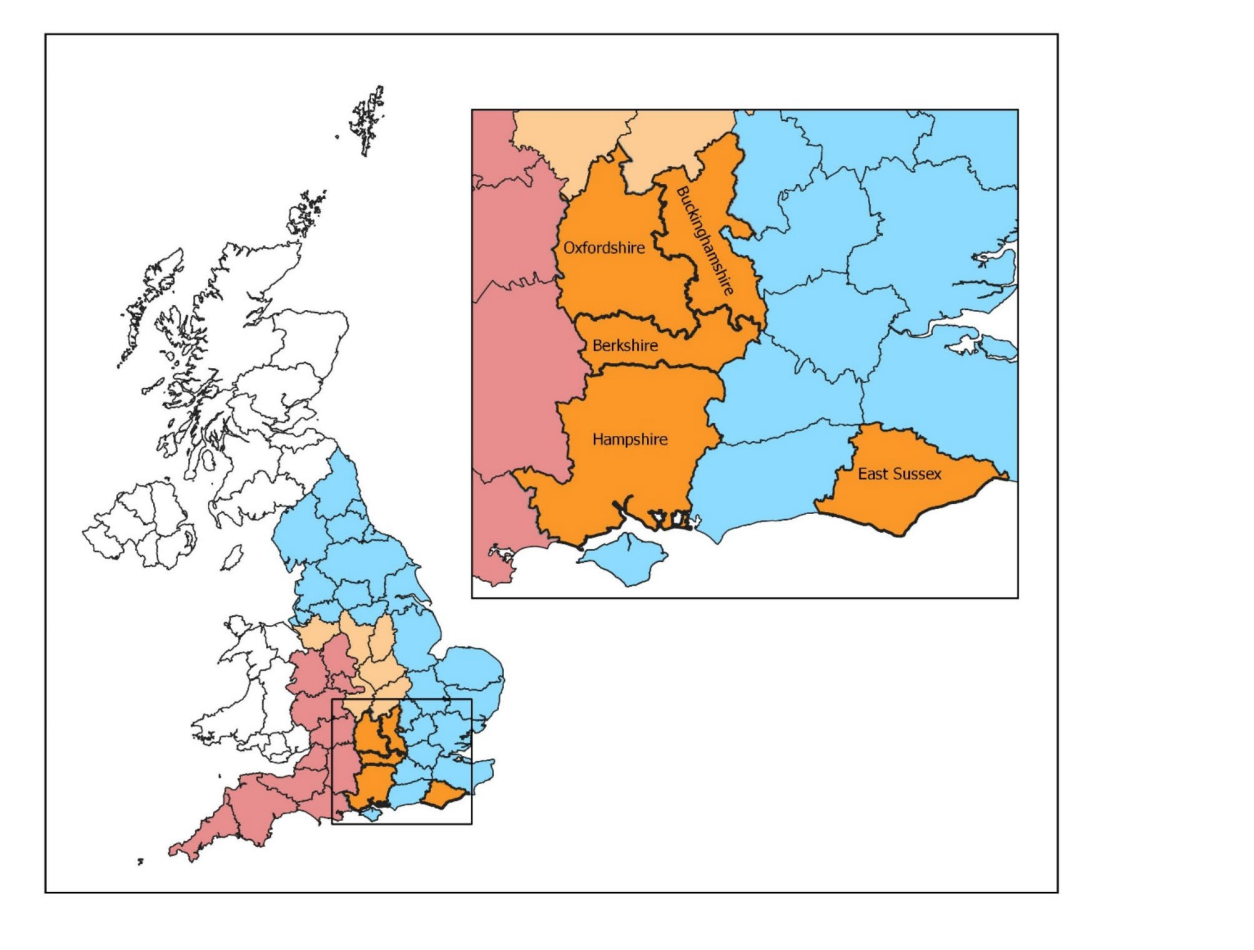
Map showing the counties of England coloured by perceived bTB risk; red indicates the high-risk area, orange the edge area, and blue the low-risk area. The studied counties are outlined in bold and non-faded. The insert shows the counties of the southern Edge Area which were the focus of the present study. Mapping software: Mapping software: QGIS version 3.36.1, QGIS Association ( http://www.qgis.org).

English counties are categorised by their historic prevalence and epidemiology of bTB infection in cattle (see Fig. 1). In the high-risk area (HRA), which encompasses the southwest of the country, the disease is endemic in cattle and extensive disease surveillance is in place, including standard six-monthly herd testing and pre-movement testing. Surrounding the HRA is the edge area (EA), where bTB prevalence has been historically lower than the HRA but into which the epidemic is expanding, resulting in highly variable disease prevalence and either six-monthly or annual cattle testing, in addition to pre-movement testing. Though not contiguous with the rest of the EA, East Sussex is included within this zone due to the prevalence of bTB in the county. Finally, in the low-risk area (LRA) the incidence of bTB in cattle has been historically low and stable, with most cattle herds routinely tested for TB every four years, supplemented by targeted enhanced testing of certain herds and mandatory post-movement testing of cattle introduced from the rest of England and Wales^12^.

While cattle movements undoubtedly make a substantial contribution to the spread and persistence of bTB in herds across the three risk areas, the presence of infection in the primary wildlife reservoir, the European badger (*Meles meles*) adds further complexity to disease control^13–15^. Infectious badgers can excrete *M. bovis* in their saliva, urine, faeces or from ruptured abscesses, presenting a hazard to cattle through indirect transmission, and potentially via direct transmission in cattle housing^16–19^. During the Randomised Badger Culling Trial (RBCT, 1998–2005), 15.9% of culled badgers tested positive by culture or by having lesions containing acid-fast organisms^20^. There was a demonstrable association between the proportion of positive badgers and cattle TB incidents at a distance of 1–2 km^20,21^. In addition, spoligotyping and multi-locus variable number tandem repeat analyses (MLVA) have demonstrated the co-localisation of related *M. bovis* isolates from badgers and cattle^21–24^. Whole genome sequencing (WGS) has further evidenced the link between infections in cattle and badgers, with highly related isolates being cultured from sympatric populations indicating recent and bi-directional transmission^25–27^. In English areas of high cattle incidence, efforts to reduce transmission risks from wildlife have included licenced culls of badgers, with over 53,000 animals culled during the years 2022 to 2023^20,22,28–32^.

For the purposes of surveillance, found-dead surveys are the most viable, practicable and consistent approach for collecting a sufficiently large sample of badgers (typically the results of road traffic accidents, RTAs) for investigating infection prevalence at a broad geographic scale (e.g., county-level). This approach was extensively validated alongside the RBCT and found to be a reliable proxy for *M. bovis* infection prevalence within the sampled population^15^. Since then, it has been employed across Wales^23,33^, Northern Ireland^24^ and in the county of Cheshire to investigate an emerging cluster of TB in cattle^34^. In addition, an investigation of bTB in found dead badgers in the northern EA is of relevance to the design of the current study, which this study was designed to complement^35^. The northern EA study took place in 2016–2017 and identified a headline MTC infection rate in badgers of 8.3% (range: 4–15%), and common areas of high-prevalence in cattle and badgers as also reported in other studies^23,24,33,36^.

Effective control of bTB in cattle at the edge of the endemic area requires a better understanding of the potential role of badgers in the spread and perpetuation of infection in this transitional zone. Here we present the results of an investigation into the prevalence of *M. bovis* in found-dead badgers in the southern EA, undertaken between 1st of April 2021 and the 30th of April 2023. Our results are interpreted alongside contemporaneous data on infection in cattle, gathered during routine surveillance and monitoring, and the results of the earlier study undertaken in the northern EA^35^.

## Results

### Badger carcasses

In total, 525 badger carcasses were collected from across the five counties of the southern EA. Of these, 428 (81.5%) were deemed suitable for examination and tissue sampling, and four (0.8%) were excluded due to not being collected within the study area. RTAs accounted for 84.3% of submissions (*n* = 443), sixteen (3.0%) were found dead elsewhere and were typically emaciated and in poor condition, whilst the causes of death could not be determined in the remainder, usually owing to the degree of carcass decomposition. The number of carcasses submitted varied by county (χ^2^ = 46.0, df = 4, *P* < 0.0001), with Oxfordshire yielding the highest number (*n* = 138) and Berkshire providing the fewest (*n* = 49) (Table 2). The majority of carcasses were collected by farmers and farming groups (*n* = 231, 44.0%), followed by conservation groups (*n* = 132, 25.1%), veterinarians and their affiliates (*n* = 85, 16.2%), government agencies or local authorities (*n* = 18, 3.4%), and miscellaneous other groups or individuals (*n* = 59, 11.2%).

**Fig. 2:**
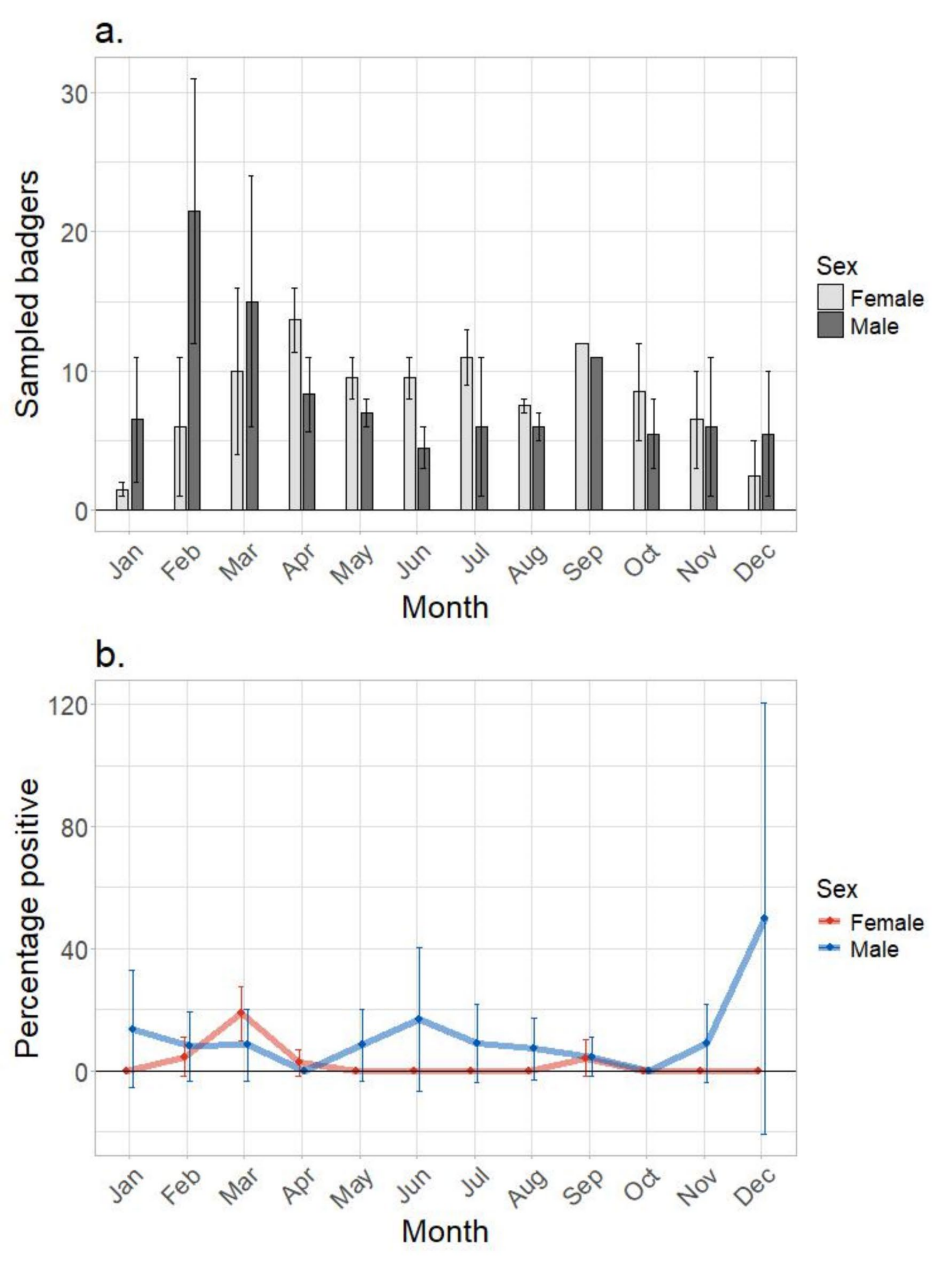
The mean number of badger carcasses collected per month by sex (**a**) and the mean percentage of carcasses testing positive for *M. bovis* by month and sex (**b**). Error bars show the standard error around the mean.

Of the carcasses submitted for which the sex was determined (*n* = 443), 226 (51.0%) were males and 217 (49.0%) were females. No temporal variation was observed in the average number of carcasses submitted by month. Comparisons between the submissions of male and female carcasses with reference to month had to account for variable sample sizes, the low number of repeat data and the unequal number of repeats. On average, there were fewer submissions of males across the calendar year, with the exception of December – March (Fig. 2). However, only in April were significantly more females submitted than males (t = 9.5, df = 2, *P* ≤ 0.01). The majority of carcasses were of adult badgers (estimated to be over 1 year old), with only 52/525 (9.9%) of those collected and 43/444 (9.7%) of those examined recorded as cubs.

### *Mycobacterium Bovis* infection in surveyed badgers

Across the southern EA, 31/428 (7.2%, 95% CI: 5.0-9.7%) badger carcasses tested positive for MTC bacteria by culture, and subsequently confirmed using IS*6110* PCR for MTC, and *hsp65* sequencing for presumptive identification of *M. bovis* (see methods for details). Of these, 28 were confirmed as *M. bovis* by WGS giving an apparent prevalence of 6.5% (95% CI: 4.4–8.9%). Despite the low number of positive badgers, and the aforementioned study design, efforts were made to examine factors that increased the likelihood of positive carcasses being detected. The prevalence of infected badgers varied significantly by county (χ^2^ = 12.7, df = 4, *P* ≤ 0.01), ranging from 1.1% in Hampshire to 13.0% in Oxfordshire (Table 2). Additionally, the detection of positive carcasses varied with the time of year, being most likely in March (OR (95% CI) = 10.58 (1.794–20.14), *P* < 0.05), and least likely in October (not significant) (Fig. 2).

Conservation/vaccination-affiliated collectors provided a higher percentage of positive submissions than other collectors (Table 1). However, underlying geographic bias skewed this figure as this group submitted significant numbers from counties with the highest TB burden; 43.2% (57/132) of their submissions were from Oxfordshire and 25.8% (34/132) were from Berkshire. In contrast, badgers submitted by farming-affiliated collectors were more evenly distributed across the study area with percentage positivity estimates skewed by the relatively smaller contribution this group made in the counties of highest TB burden. Collector affiliation was not thought to influence the likelihood of a positive submission.

**Table 1:** Summary of *M. bovis* positive carcasses submitted by self-identified affiliation.

Type	Submissions (*N*)	Positive (*n*)	Positive (%)
Farming	231	9	3.90
Conservation/vaccination	132	12	9.09
Veterinary	84	4	4.76
Animal cremation	21	1	4.76
Other/not provided	21	1	4.76
Government dept/agency	17	0	0.00
Research	7	1	14.3
Country sport	4	0	0.00
Contractor	2	0	0.00
Education	1	0	0.00
Pest control	1	0	0.00

Submission values include badgers which were not subjected to post-mortem due to being in very poor condition.

All of the confirmed positives were adults (28/384 confirmed adults, 7.3%, 95% CI: 4.9–10.4%) and the prevalence of infection was more than 3 times higher in male badgers than in females (χ^2^ = 8.3, df = 1, *P* < 0.01), with 22/215 male badgers testing positive (10.2%, 95% CI: 6.5–15.1%) and 6/210 females (2.9%, 95% CI: 1.1–6.1%). The distances between any given positive badger and its nearest positive neighbour were significantly smaller than those between any negative badger and their nearest positive neighbour (W = 4193, *P* < 0.05). The median distance from a positive badger to the nearest other positive badger was 6.1 km (interquartile range, IQR: 2.4–11.6 km), whilst negative badgers were on average 8.4 km (IQR: 5.1–16.5 km) from their nearest positive neighbour.

**Fig. 3:**
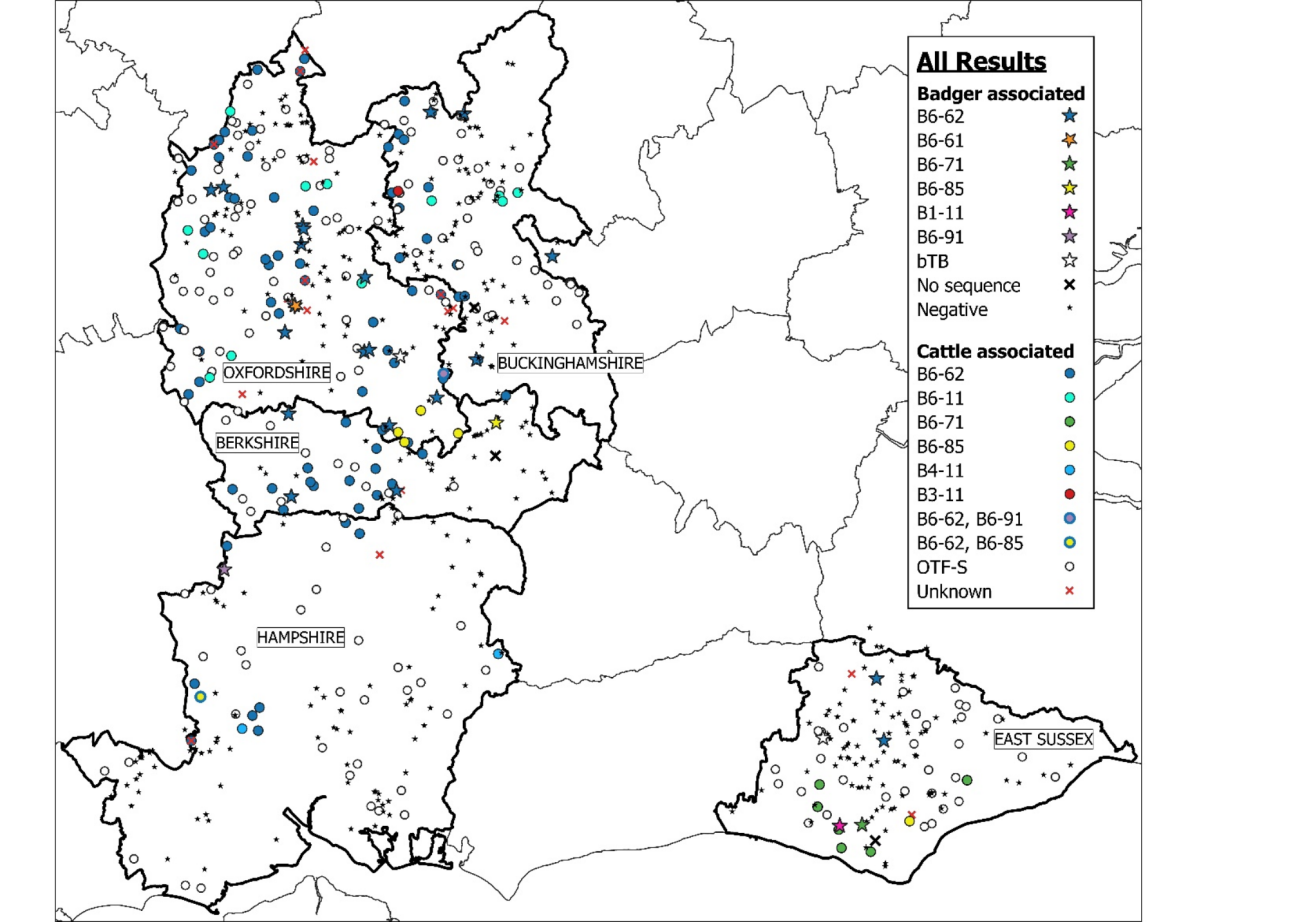
The spatial distribution of the sampled badger carcasses, and herd level TB test results during the study period (1st April 2021–30th April 2023). Stars or black crosses indicate badger carcass test results; filled circles or red crosses indicate cattle herds where Officially bTB Free status was withdrawn (OTF-W) due to a TB incident (breakdown) with at least one lesion and/or M. bovis culture-positive animal removed, while unfilled circles indicate suspended herd status (OTF-S). Mapping software: QGIS version 3.36.1, QGIS Association (http://www.qgis.org).

Six *M. bovis* clades were identified amongst the isolates obtained from confirmed positive badger carcasses. Of these, B6-62 was the most common, representing 21/28 (75.0%) of *M. bovis* confirmed isolates. The remaining clades (B6-61, B6-71, B6-85, B6-91, and B1-11) were each detected only once while two isolates (7.1%) were not identified to clade level (Fig. 3). The distance to the nearest badger with the same strain (where available) had a mean of 7.9 km (95% CI: 5.4–10.7 km) while the distance to the nearest badger with a different strain was 17.4 km (95% CI: 12.7–22.0 km). Statistical analysis identified a significant difference between the two distributions (t = -4.57, df = 20, *P* < 0.001).

### *Mycobacterium bovis* in cattle

During the study period, there was an average of 2639 (range: 2606–2711) active herds in the southern EA per annum. Of these, 277 herds (10.5%) experienced at least one TB breakdown (i.e. detection of at least one TB test positive animal and/or laboratory-positive case at routine slaughter). During the study period, a total of 308 breakdowns occurred in the study area (including 31 repeat breakdowns) of which 132 (42.9%) had their ‘Officially bTB Free’ (OTF) status withdrawn (OTF-W), 174 (56.5%) had their OTF status suspended (OTF-S) and the remainder were unclassified. Of the breakdowns involving at least one animal with visible tuberculous lesions or positive culture (OTF-W), WGS clades were identified in 84.1% (*n* = 111) of incidents, meaning that amongst all incidents of OTF status being suspended or withdrawn a clade was identified in 36.0% of cases.

The proportion of herds with reactor cattle during the period of study varied by county (χ^2^ = 13.7, df = 4, *P* < 0.01); Oxfordshire herds were the most likely to have a TB test reactor detected with 20.9% of herds experiencing a breakdown during the study, compared to only 5.0% of herds in Hampshire (Table 2). Of those herds where a clade associated reactor was identified (*n* = 111), the most common *M. bovis* clade was B6-62 which was found in 83 incidents (74.8%), followed by B6-11 in twelve incidents (10.8%).

**Table 2:** County level demography and TB status of badger carcasses submitted during the study period (1st April 2021–30th April 2023), and data on the number of active herds per county and their breakdown status (1st April 2021–30th April 2023).

		County				
		Oxfordshire	East Sussex	Buckinghamshire	Hampshire	Berkshire
Badgers^a^	Carcasses submitted	138	128	99	107	49
	Carcasses sampled^b^	108 (78.2%)	103 (80.4%)	85 (85.9%)	88 (82.2%)	44 (89.8%)
	Carcass density (per km^2^)	0.0415	0.0575	0.0454	0.0239	0.0349
	Male (%)	46.4	51.4	48.9	55.1	57.8
	Female (%)	54.6	48.6	51.1	44.9	42.2
	Adults (%)	88.0	96.1	97.6	94.3	95.5
	Juveniles (%)	12.0	3.9	2.4	5.7	4.5
	*M. bovis* positive	14 (13.0%)	5 (4.9%)	4 (4.7%)	1 (1.1%)	4 (9.1%)
	*M. bovis positive* (per km^2^)	0.00537	0.00279	0.00213	0.00027	0.00317
Cattle^c^	Mean active herds (range)	526 (509–545)	590 (584–603)	475 (467–494)	837 (820–857)	211 (208–215)
	Mean active herd density (per km^2^)	0.202	0.329	0.253	0.228	0.167
	Number of herds with breakdown^d^	110	41	53	42	31
	Percentage of herds with breakdown	20.9	6.9	11.2	5.0	14.7
	Density of breakdowns (per km^2^)	0.0422	0.0229	0.0283	0.0114	0.0246

^a^Note that unsampled carcasses were excluded from the analyses. ^b^One badger was excluded from these analyses as the spatial data indicated it was located significantly beyond the study boundary. ^c^Data is given at the herd level and analyses conducted using the mean number of active herds during the study period. ^d^Estimated as the number of herds that were non-OTF during the study period; repeat breakdowns are not included.

### Comparison between badgers and cattle

**Fig. 4:**
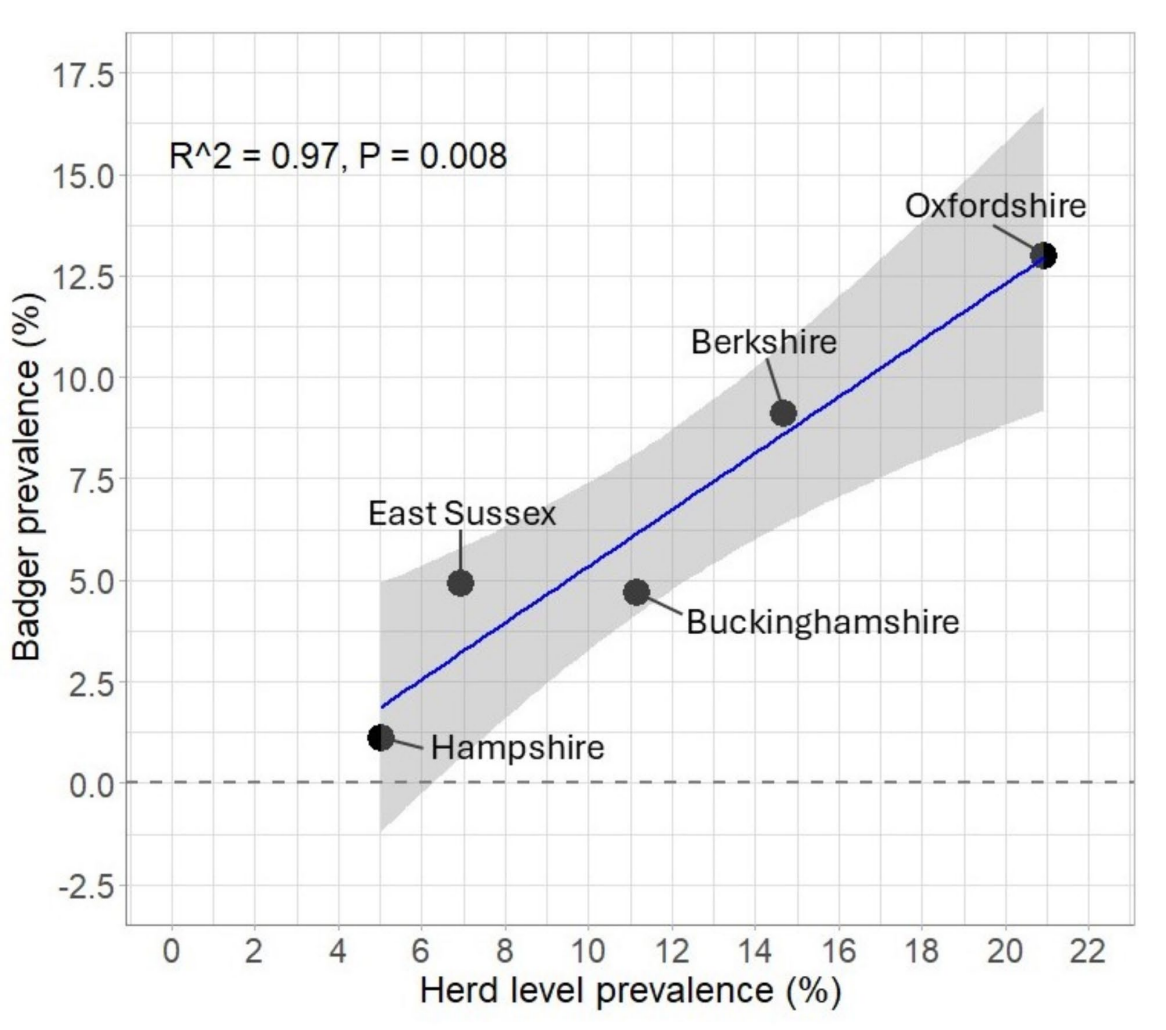
County-level comparison of the percentage of *M. bovis* infected badgers (detected by culture and WGS) and infected cattle herds (as determined by the single intradermal comparative cervical tuberculin skin test (SICCT)) in the Southern Edge Area Study (April 2021–April 2023). Repeat breakdowns are excluded from these summary data.

A statistically significant, positive relationship was identified between the percentage of positive badger carcasses and the herd level prevalence (defined as the percentage of herds that experienced at least one breakdown during the study period) at the county level (Fig. 4; Pearson’s r(3) = 0.97 (95% CI: 0.82-1.0), t(3) = 10.9, *P* < 0.01).

The distances between badger carcasses and cattle infected with the same and different clades were analysed. The two East Sussex B6-62 infected badgers were removed from the analyses as outliers, due to the non-detection of the clade in local herds and because this county was not contiguous with any other EA county. Overall, the distance from a positive badger to a herd with the same clade was 3.6 km (IQR: 2.9–5.6 km) while the mean distance to the nearest herd infected with a different clade was 9.9 km (95% CI: 7.6–12.2 km). Badgers were significantly more likely to be found near herds infected with the same clade, than to be found near herds infected with different clades (V = 41, *P* < 0.01).

### Non-tuberculous mycobacteria (NTM)

Fifteen putative species of non-tuberculous mycobacteria (NTM) were detected in 30 badgers (apparent prevalence 7.0%, 95% CI: 5.1–9.4%). *M. avium* was the most prevalent NTM with an apparent prevalence of 2.6% (95% CI: 1.3–4.5%, 11/429), followed by *M. vaccae* with an apparent prevalence of 1.2% (95% CI: 0.4–2.7%, 5/429); some isolates were not fully identified by this method. More males tested positive for *M. bovis* (22/215, 10.2%) than for NTM (16/215, 7.4%) and more females tested positive for NTM (13/210, 6.2%) than for *M. bovis* (6/210, 2.9%), although neither difference was significant. One unsexed badger was also positive for NTM.

## Discussion

*Mycobacterium bovis* infection in badgers collected from the southern EA was spatially heterogeneous, with the county level apparent prevalence ranging from 1.1% in Hampshire to 13.0% in Oxfordshire. This concurs with findings from a similar study of found dead badgers in the northern EA where county-level prevalence ranged from 4–5 to 15%^35^. This marked spatial heterogeneity of the disease has also been demonstrated in Wales^23^ and the Republic of Ireland^36^. Our study wide prevalence estimate of 6.5% (95% CI: 4.4–8.9%) is also similar to that of other studies in the northern EA (8.3%, 95% CI: 6.4–11.0%) and Wales (7.3%, 95% CI: 5.6–9.5%) which are contiguous with England’s HRA^23,35^. Despite nearly two decades of separation, the overall apparent prevalence remains lower than that of the HRA (2002–2005), in which 16.6% of culled badgers and 15% of RTA badgers were positive for *M. bovis*^15^ demonstrating the slow expansion of the disease. However, direct comparisons between studies can be problematic owing to minor methodological differences. For example, the current study used WGS to confirm *M. bovis* isolation whilst Swift et al. (2021) used IS*6110* to confirm only to the MTC level, and Schroeder et al.. (2020) used spoligotyping. WGS excluded three samples that were identified as MTC members by IS*6110* PCR and *hsp65* sequencing (putative *M. bovis* due to the host species), reducing the overall apparent prevalence from 7.2% to the reported 6.5%. This highlights that differing methodologies can produce slight differences in prevalence estimates.

The prevalence of *M. bovis* in badgers was positively correlated with the number of incident cases in cattle at the county level, consistent with findings in the northern Edge Area, and in endemic areas of England, Wales and the Republic of Ireland (RoI)^22,23,33,35,36^. At the clade level, infected badgers were geographically closer to herds infected with the same clade than those with different clades, as previously shown in the HRA using spoligotyping^21^ and in Northern Ireland using MLVA^24^. Clustering of positive badgers was also evident as has been observed in the RoI^36^ and England’s HRA^37^.

The ratio of male to female badgers in the present study was circa 1:1, similar to that observed in other found-dead studies^33,35,38^ and as identified in capture-mark-recapture studies of badger populations^39–41^. The prevalence of confirmed *M. bovis* infection in male badgers in the present study was higher than in females, consistent with previous work showing that males are more likely to become infected and experience more rapid disease progression, thus increasing the likelihood of yielding a positive culture at post-mortem examination PME^42,43^. It is also hypothesised that immunological differences may enhance male susceptibility to disease^43^.

The present study resulted in a lower percentage of sampled juvenile badgers (9.9%) compared to other found dead surveys using similar collection methods (c. 30% ^34,35^ and population studies (c. 20–30%)^41,44,45^. As TB infection is more often observed in adult badgers than in juveniles, the high proportion of adults in the present study is likely to have inflated overall population-level prevalence^20,46,47^. The reasons for the low percentage of juveniles in our sample of carcasses are not clear, but could potentially reflect poor survival related to unfavourable conditions^48^. For example, April 2021 and 2022 and the summer of 2021 were unusually dry^49,50^ which is likely to have reduced food availability^51,52^ and impacted on cub survival in particular^48^.

Routine WGS of *M. bovis* requires the culture of bacterial isolates from infected individuals. However, the success of culture can be limited by early-stage infection^53,54^, the metabolic state of the bacteria^55,56^, the slow growth rate of *M. bovis*^57^ and the potential for overgrowth by competing microorganisms^58^; it is therefore highly probable that the apparent prevalence given here is an underestimation of the true prevalence. Furthermore, the current routine approach of sequencing a single isolate per badger or entire cattle herd means that if multiple infections with different strains or clades were present then they would not be detected. Despite these limitations, six clades were cultured from badger carcasses during the present study, of which B6-62 was the most common, being identified in 4/5 counties. Two badgers collected from East Sussex were infected with B6-62, outside the putative home range of the clade, and at that time the clade was undetected in the county’s cattle^59^. The probability that these two samples were similarly mislabelled seems low given that both the examination of carcasses and subsequent sequencing of isolates were carried out several weeks apart. Furthermore, B6-62 has recently been detected in the county’s cattle, within two herds which were OTF-S during the present study, and therefore may have been present for some time^60^. Two further clades which were identified during the study (B1-11, B6-85) were also isolated from badgers in areas which were well beyond their typical home range, and one nationally rare isolate with no designated home range was detected in Oxfordshire (B6-61)^59^. While B6-85 was identified in a badger found in close proximity to cattle herd breakdowns associated with that clade, the same was not true for B1-11 and B6-61. The identification of *M. bovis* clades in badgers that are distinct from those detected over the same period in cattle could arise as a result of historic spillover from cattle into badgers, or the limited sensitivity of *M. bovis* surveillance in cattle, as only 36% of breakdowns yielded sequenceable isolates across the study area. Previous evidence has demonstrated the contemporaneous circulation of different strains within badgers and cattle, showing the value in surveying both species to entirely evaluate the disease landscape^25^.

The apparent prevalence of NTM in this study was 7.0%, far greater than a previously reported value of 0.9% (424/45,705) from archived UK cull data (recorded as *M. avium* and other mycobacteria), but similar to that reported in Spain from a small opportunistic study^61^. Unlike *M. bovis*, NTMs were distributed relatively evenly between the sexes, likely related to the environmental nature of these bacteria contributing to even exposure risks. Also, in contrast to *M. bovis*, the likelihood of detectable NTM infection did not increase with age, though the small number of juveniles in this study likely limited our ability to detect any such effects.

Estimates of prevalence from studies such as ours should be treated with caution as biases can arise as a result of the relatively small sample sizes, the unknown absolute population size, local clustering of infection, potential behavioural correlates of susceptibility to becoming a RTA, and the limitations of the diagnostic methods employed. However, the sampling approach and culture-based methodology in the present study are broadly consistent with those shared by several other badger RTA surveys, allowing for some level of comparison whilst recognising the known limitations^23,33–35^. Furthermore, the collection of found dead badgers provides a geographically broad estimate of prevalence, which would otherwise be unachievable, and the collection of epidemiologically valuable WGS data to assist in disease tracking. There were underlying concerns that the apparent prevalence may be susceptible to collector bias, but when considered alongside other factors such as the county level prevalence, it was thought unlikely that the submissions were biased – with conservation/vaccination-affiliated collectors submitting a greater percentage of positive carcasses than farming affiliated collectors.

To conclude, the present study has provided the first estimates of *M. bovis* prevalence in badger populations in the southern EA. In addition to identifying county-level heterogeneity, the use of WGS has added significant value to the surveillance data available for cattle by uncovering additional clades in badgers that were not detected in local herds and has confirmed infection in wildlife where it was previously unstudied. This study has provided further evidence for the link between infection in badgers and cattle at the edge of the area of TB endemism in England. Further investigation of WGS data from this area may shed light on the relative importance of cattle or badgers in driving herd breakdowns and whether (as seems likely) cattle movements may have seeded infection in both local cattle and badger populations. This may, in some locations, facilitate the circulation of infection in the local badger population and risk spill-back to cattle. Understanding the proximal drivers of infection in cattle across the EA may inform management options which could include enhanced cattle measures, badger vaccination and targeted badger culling. In some parts of the EA it may not be too late to prevent infection spilling over into this wildlife host before it becomes endemic in both populations.

## Materials and methods

### Carcass collection and storage

A network of collectors was established across the five counties of the southern Edge Area with the aim of collecting 100 carcasses from each county. A dedicated phoneline and e-mail address were used for communications, and the study was promoted at events, through veterinarians, and on the TB Hub website. Interested parties were provided with instructions and kits for the safe collection of found-dead badgers as well as a small financial reward. Collectors placed carcasses in three double thickness PVC bags and sealed them with pre-labelled tags. Useful metadata was recorded on the submission form by the collector, including the date and time, tag identification code, location, type of submission (e.g., road traffic accident) and any overnight storage conditions if the courier was unable to collect the same day. Carcasses were not frozen, but when storage was required, collectors were asked to place in a chiller if available, or else in a cool and dry area. If the courier was unable to transport the carcass to the PME lab (University of Nottingham, Sutton Bonington Campus) the same day, then it would be stored in a chiller overnight or over the weekend until it could be safely received. The median time between carcass collection and PME was 3 days (range: 0–12). Carcass collection took place from April 2021 to April 2023 inclusive, with collections ceasing at the county level once 100 carcasses had been sampled.

### Post-mortem examination

Carcasses were deemed suitable for PME if they had not suffered significant damage, were not autolysed, and arrived with the form linking them to a location. The sex, weight, length, age, (adult or juvenile), estimated from dentition, size, and pelage, condition as inferred from weight and body fat, and probable cause of death were recorded. In some cases, these data are not recorded due to the condition of the carcass, but sampling was possible, thus test data exists that cannot be related to certain population demographics. The tissue sampling protocol for isolation of mycobacteria followed that of the Northern Edge Study^35^, based on the protocols used by ^33^ and ^36^, modified by^34^. Carcasses were examined externally and internally for TB-like lesions, and if present these were harvested for tissue processing and bacterial culture. Selected lymph nodes and lung tissue were harvested and pooled (see Table 3). Harvested tissues were stored at 4 °C for up to 48 h before processing for bacterial culture.

**Table 3:** Tissue pools harvested from badger carcasses for microbiological culture.

Tissue pool	Tissues
Lesions	Individual samples from each gross lesion
Head and neck	Right and left mandibular, parotid, and retropharyngeal lymph nodes
Thorax	Anterior and posterior mesenteric and left and right bronchial lymph nodes, and apical lung
Abdomen	Hepatic and mesenteric lymph nodes
Carcass pool	Right and left prescapular, axillary, inguinal, and popliteal lymph nodes
Thoracic blood/fluid	Frozen for serology

## Tissue processing

Tissue processing and microbiological examination was performed in the Containment Level 3 (CL3) facility at the University of Nottingham, according to the methods used in previous studies^34,35^. Briefly, lesioned tissue or pools of target tissues were gently ground with sterile sand in phosphate buffered saline (PBS). Samples were mixed 1:1 with 5% oxalic acid and incubated at room temperature for 10 min, before 200 µL of each pool was inoculated onto Stonebrink Selective agar + PACT (BD Diagnostics) and onto Middlebrook 7H11 slopes supplemented by PANTA (BD Diagnostics). Media were incubated at 37 °C for a minimum of 12 weeks with checking at regular intervals for putative mycobacterial colonies.

### Characterisation of mycobacteria

After a minimum of 12 weeks incubation, putative mycobacterial colonies were heat killed at 80 °C for 30 min. DNA was extracted by crude lysis; heat killed colonies were frozen and subsequently heated to 95 °C for 5 min in sterile distilled water (SDW), centrifuged at 13,000 x g for 3 min to remove cellular debris and the resultant supernatant aliquoted for molecular analyses.

Isolates were screened by PCR for IS*6110*, an insertion sequence unique to the MTC, and *hsp65* sequencing which also identified non-tuberculous mycobacteria (NTM). Confirmed members of the MTC were subjected to whole genome sequencing (WGS; performed at APHA laboratories, Weybridge). Sequences were compared to *M. bovis* reference sequence AF2122 for final confirmation and assigned to one of 30 lineages known to be in circulation in Great Britain (pipeline: https://github.com/APHA-CSU/btb-seq)^62^. Major groups are denoted by the initial ‘B’ number denoting ‘*bovis’* with a range of 1–6. The second number is hierarchical and demonstrates relatedness within the major group, i.e. B6-61 is more closely related to B6-62 than it is to B6-41, but all of these are more closely related than they are to another major group (e.g. B3-11)^63^.

### Cattle data

APHA conducts routine surveillance and testing of cattle herds for bTB. In the HRA and EA cattle are tested annually or six-monthly, with increased frequency of testing following detection of infection or after high-risk cattle movements. Testing is routinely conducted in the UK using the single intradermal comparative cervical tuberculin test (SICCT) with subsequent removal of reactors (animals exhibiting a positive result). Herds that are on schedule with their testing routine and have no reactors are classed as ‘Officially bTB Free’ (OTF). If at least one reactor (a bovine with one positive or two inconclusive results) was detected, then a TB incident (herd breakdown) is declared and the OTF status is suspended (OTF-S). At PME, if lesions that are typical of TB are identified in a reactor, or a positive bacterial culture or (as of March 2022) positive PCR test is obtained from a TB test reactor, or a non-reactor animal presenting with suspected tuberculous lesions at routine slaughter, then the OTF status of the affected herd is withdrawn (OTF-W). The APHA held dataset includes the breakdown identifier, herd identifier, case reference, herd location, breakdown status, the test result and WGS clade if determined. For the present study, the data for each herd was combined across the study period and any herd that had been OTF-S or OTF-W was classed as positive.

### Statistical analyses

Analyses were conducted in R version 4.2.2, with graphs generated using the ‘ggplot2’ and ‘patchwork’ packages. The ‘sf’ package was used to analyse spatial data and QGIS 3.8.1 to visualise it. Statistical comparisons of count, percentage and distance data were performed within this study; data were tested for normality using the Shapiro-Wilk test. Significance testing of count and distance data was performed using the following: students T-test for normal and non-percentage data otherwise a Chi-squared when sufficient data were available, else the Fisher’s Exact Test. Paired data was tested using a T-test for normal data or Wilcoxon Signed Rank Test when the data were not normal. Multiple comparisons were controlled using the Benjamini-Hochberg method to reduce the false discovery rate.

## Data Availability

Data from cattle and badgers will be available on figshare (10.6084/m9.figshare.27226140). Due to the sensitivity of the badger data, these locations are only available to within 1 km. For any queries regarding the dataset or its availability please contact the corresponding author.
